# GH provocative tests stimulate the growth in children with idiopathic short stature

**DOI:** 10.1007/s12020-024-03860-x

**Published:** 2024-05-15

**Authors:** Anna Tortora, Vincenzo Marotta, Giulia Izzo, Domenico Rocco, Gennaro Clemente, Mario Vitale

**Affiliations:** 1 UOC Clinica Endocrinologica e Diabetologica, AOU San Giovanni e Ruggi d’Aragona, Salerno, Italy; 2 Dipartimento di Medicina, Chirurgia e Odontoiatria, Università di Salerno, Baronissi, Salerno Italy; 3 IRPPS Consiglio Nazionale delle Ricerche, Penta di Fisciano, Salerno, Italy

**Keywords:** Growth hormone deficiency, Short stature, GH provocative tests

## Abstract

**Context:**

Growth hormone (GH) deficiency in a child with short stature is diagnosed by GH secretion provocative tests. When the test response is considered adequate, the short stature is considered idiopathic (ISS).

**Objective:**

To determine the effect of GH provocative tests on the growth rate in children with idiopathic short stature.

**Design:**

Children with short stature with a normal response to at least one GH provocative test were enrolled. Height and growth velocity were measured prior to and after stimulus tests during the follow-up.

**Methods:**

Height, mid-parental height, body weight, and body mass index were measured. The height and growth rate were converted to percentiles and Standard Deviation Scores (SDS) using reference ranges standardized by age and sex. GH provocative tests employed arginine or clonidine as secretagogues.

**Results:**

Fourty-six children of both genders were enrolled. In thirty-six children, height was measured at the time of testing and on an average time prior to and after the tests of 210 days and 180 days respectively. After testing the children displayed a 3.4-fold increase in their estimated 90-day growth rate. The median (inter-quartile range, IQR) 90 days growth of children pre-and post-tests were 0.7 (0.2–1.0) cm and 2.4 (1.7–3.1) cm respectively with a mean 3,4-fold increase (*p* < 0.0001). The median (IQR) 90 days growth of children pre- and post-tests calculated as standard deviation scores (SDS) were −4.0 (−5.4–−2.1) SDS and 0.1 (−1.9–1.4) SDS respectively (*p* < 0.0001). Ten children with ISS were observed for about 5 months before the GH provocative tests. A small increase in the growth rate was seen only in 2 out of 10 children before testing while it increased in all of them after the tests. The difference in the median growth rate at the first and the second observation was not significant (*p* = 0.219).

**Conclusions:**

Two sequential somatotropic axis provocative tests increase the growth rate in children with idiopathic short stature. The duration of this effect is yet to be determined.

## Introduction

Growth hormone (GH) secretion, synthesized in the anterior pituitary gland, is regulated by the coordinated action of GH GH-releasing hormone (GHRH) and Somatostatin (SS) which respectively stimulates and inhibits its release [1]. GH secretion is also controlled by a complex negative feedback mechanism mediated by Insulin-like Growth Factor (IGF-1) which exerts its inhibitory action both at hypothalamic and pituitary levels [1]. Other hormones, including sex steroids, glucocorticoids, gastrointestinal peptides, and some glucometabolic pathways participate in the secretory regulation of GH which is characterized by being episodic and with night-time peak [1, 2]. IGF-1 is the main anabolic mediator of somatotropic activity, responsible of GH-induced cell proliferation [3].

GH deficiency (GHD) is a clinical disorder that is characterized by pathological short stature in the child, altered body composition, impaired psychological well-being, and reduced quality of life [4, 5]. These alterations are almost always reversible after recombinant human GH (rhGH) administration, which is currently the only accepted treatment for patients with GHD. Although it is difficult to extract an exact prevalence rate of GH deficiency, it is considered a rare cause of short stature, with an incidence of about 1/4000 born alive [6].

GHD diagnosis during developmental age is based upon clinical evaluation associated with laboratory assessment of the GH/IGF-1 axis and radiological investigations [7]. GH physiological values are pulsatile and low and the available tests have a poor sensitivity, making serum GH dosage insufficient to document a lack of secretion. Therefore, in the absence of congenital forms and/or malformations of the adenohypophysis, peduncle, and/or neurohypophysis, GHD diagnosis requires GH secretion provocative tests.

Secretory dysfunction is confirmed when the GH peak does not reach the established cut-off in at least two different stimulus tests performed on two different days [7]. Several provocative tests have been proposed in the last decades, namely GHRH, insulin hypoglycaemia, clonidine, arginine, L-dopa, and glucagon [8].

The Italian Medicines Agency (AIFA) established with the last Note 39, a cut-off value of GH < 8 ng/mL in two provocative tests performed on different days to diagnose GHD in children. Only the children with GHD according to the Note 39 have access to rhGH therapy reimbursement. Consequently, several children with short stature and/or pathological deflection of growth velocity are tested for suspected GHD but are not treated with rhGH because they have a response equal to or above the cut-off of 8 ng/mL to one of the two stimulus tests and are diagnosed to have idiopathic short stature (ISS).

We followed by repeated stature measurements over time, children with pathological short stature tested for suspected GH deficiency, who responded well to GH provocative tests, and as a consequence were diagnosed to have ISS and were not treated with rhGH.

## Patients and methods

Male and female children, suspected of GH deficiency but with normal response to two GH provocative tests, were selected. Auxological data (height and growth velocity) were recorded pre and post-stimulus tests and during subsequent follow-ups over time. The study protocol was approved by the institution’s ethics committee (Comitato Etico Campania Sud), and all the participating parents gave their written informed consent.

### Subjects

Children of both genders, with pathological short stature (*h* < 3°percentile) and/or annual growth rate <−2 SDS were tested with two GH provocative tests performed on different days by using arginine or clonidine. For children with GH peak ≥ 8 ng/mL at least one provocative test entered the study if previously they had been diagnosed endocrinopathies, other organ dysfunction, autoimmune diseases, genetic syndrome, or current drug therapies were excluded. All children enrolled were adequate for gestational age at birth and had normal body proportions at the time of enrolment; moreover, all patients were prepubertal and during the follow up remained at the same stage according to the Tanner scale.

### Height and growth rate measurements

Monthly, the body weight was measured with a standard balance while the height with a standard stadiometer with a variability of 0.1 kg and 0.1 cm, respectively. Inter- and intra-operator variability was 0.3 cm. Values for height and weight were converted to percentiles and Standard Deviation Scores (SDS) using reference ranges standardized for Italian children [9]. The body mass index (BMI) was calculated as the ratio between weight in kg and height in square metres (kg/m2). The mid-parental height (MPH), expressed in cm, was calculated according to Tanner’s method: boys: (father’s height + 13 + mother’s height)/2; girls (father’s height − 13 + mother’s height)/2. The pubertal stage was assessed pre and post-stimulus test at the time of height measurement, biochemically by measurement of FSH, LH, total testosterone (in males), 17-B-estradiol (in females), and clinically according to the Tanner stages; specifically, for females with breast development and for males with testes volume checked using Prader’s orchidometer [10].

Growth velocity was assessed by the Italian Society of Pediatric Endocrinology (SIEDP) calculator which converted its value to SDS using reference ranges standardized for age and sex (version 0011, 2019).

At baseline, before GH provocative tests, all subject’s height, weight, BMI, and MPH were recorded and personal and family histories were investigated. Furthermore, in addition to general blood tests, to exclude other pituitary deficiencies and to monitor pubertal development, thyroid stimulating hormone, free thyroxine and triiodothyronine, adrenocorticotropic hormone, cortisol, follicle-stimulating hormone, luteinizing hormone, and gonadal steroid hormones were assessed.

### GH provocative tests

IGF-1 level was detected using a double solid-phase chemiluminescent immunometric assay while GH was measured using a simultaneous immuno-enzymatic assay.

GH secretion was determined by using arginine or clonidine as secretagogues; in particular, after overnight fasting, an intravenous catheter was administrated in the antecubital vein to infuse, in 30 min, arginine (arginine hydrochloride, 30% solution, S.a.l.f. Spa, Bergamo, Italy) at a dose of 0.5 g/kg (maximum 30 g); clonidine (Catapresan, Boehringer, Germany), instead, was administrated orally at a dose of 0.1 mg/m^2^. Blood pressure was strictly monitored during the whole procedure and blood samples were taken at baseline, 30, 60, 90, and 120 min after stimulus administration. All children had both tests on different days at one-week intervals.

According to current indications of the Italian Medicines Agency (AIFA) established with the last Note 39, a cut-off value of GH < 8 ng/mL, in two provocative tests performed on different days, was considered subnormal to diagnose GHD in children.

### Statistical analysis

Data are presented as absolute value or median and mean ± standard deviation or quartiles. Normal distribution was assessed by the Shapiro–Wilk statistic. Normally distributed variables were compared using the paired T-Student test, and non-normally distributed variables by the Wilcoxon signed-ranks test. *p* values < 0.05 were considered significant. Data analyses were conducted with SPSS Statistics version 26 (IBM Corp. Armonk, N.Y., USA).

## Results

Thirty-six children, 17 females, and 19 males, of average age 11 years and 2 months (range 8.8─13.8) were tested for GHD with two GH provocative tests. All the patients displayed a GH equal to or above 8 ng/mL and did not enter the treatment program with recombinant human growth hormone (rhGH) (Table 1). According to the parents survey it was established that during the follow-up the children’s habits and diet remained unchanged. All patients remained pre-pubertal before and after (180 days) the stimulus tests as demonstrated by both FSH, LH, total testosterone, and 17-beta estradiol levels and Tanner stage.

**Table 1:** Features of enrolled children at test time

Age (years) (range, mean ± SD)	8.8─13.8 11 years, 2 months ± 2
Gender	19 males, 17 females
Height (cm, mean ± SD)	130.8 ± 11.1
Height (SDS)	−2.0 ± 0.5
MPH males (cm)	172.4 ± 4.2
MPH males (SDS)	− 0,63
MPH females (SDS)	−0,73
MPH females (cm)	158.3 ± 6.2
BMI (Kg/m^2^)	17.9 ± 3.1
BMI SDS	+ 0.125 ± 1.2

**Fig. 1:**
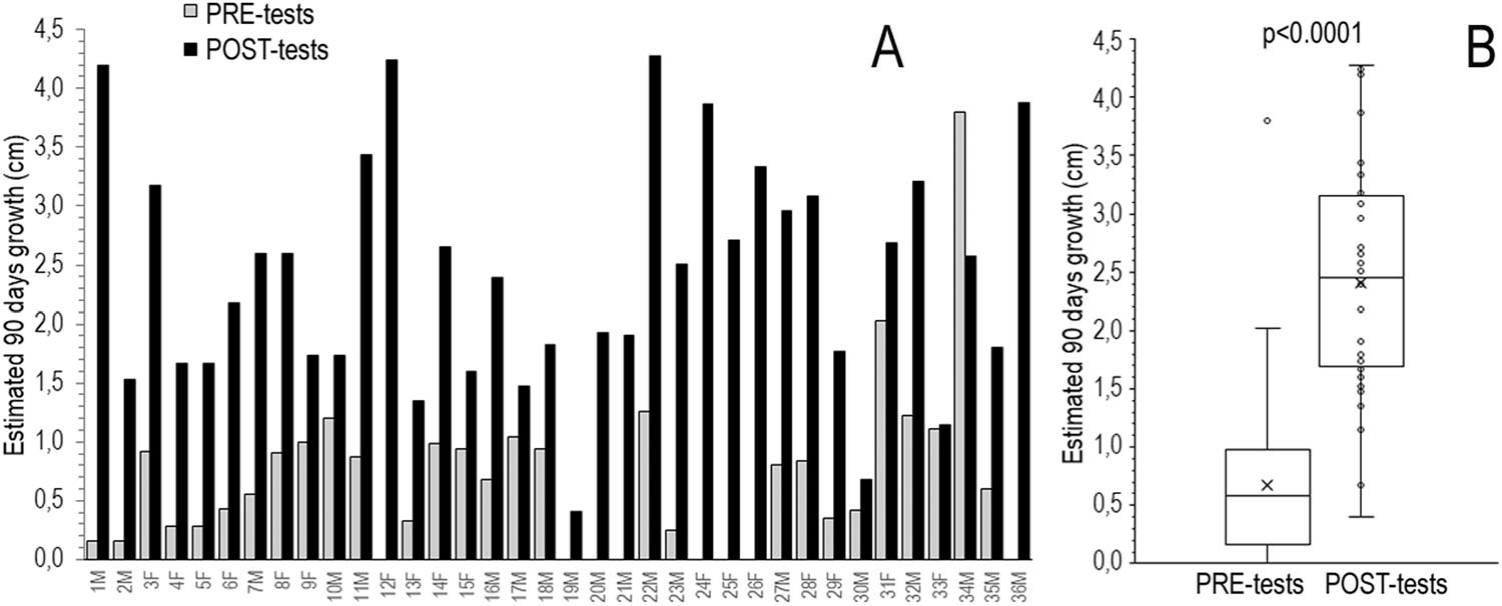
Effect of GH stimulation tests on the growth of 36 children with ISS. **A** individual estimated 90 days of growth before test time (PRE-tests) and after (POST-tests). **B** mean (x), median, quartiles, and individual values of growth. Variables were compared using Wilcoxon’s signed-rank test. Median, mean, SD, Q1, Q3 pre-test and post-test respectively: 0.7, 0.6, 0.2, 1.0, 0.96 and 2.4, 2.4, 1, 1.72, 3.11

**Fig. 2:**
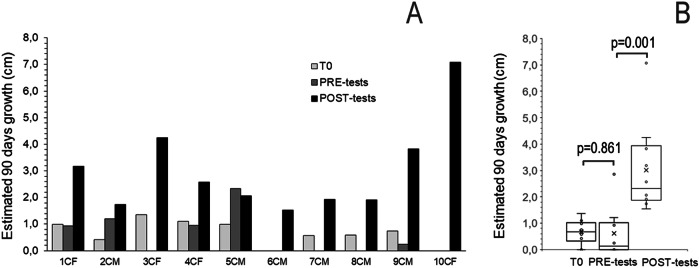
Growth of children with ISS before GH stimulation tests. **A** individual estimated 90 days of growth at the first observation time (T0), at the second observation before tests (PRE-tests), and after tests (POST-tests). **B** mean (x), median, quartiles, and individual values of growth. Variables were compared using the Wilcoxon’s signed-rank test

The pre-tests children growth rate’s value was normalized to 90 days and was determined by measuring their height at test time and at an average time of 210 days (range 43–473, SD ± 153) before. The measurements were repeated at an average time of 180 days (range 65–598, SD ± 110) after the tests. All children, both females and males displayed an increase of their growth after the tests (Fig. 1A). The median (inter-quartile range, IQR) 90 days growth of children pre- and post-tests were 0.7 (0.2–1.0) cm and 2.4 (1.7–3.1) cm respectively with a mean 3, 4-fold increase (*p* < 0.0001) (Fig. 1B). Furthermore, among our patients only two were affected by delayed puberty, both males and with a similar increase in growth velocity after the stimulus test. Due to the lockdown during the COVID-19 pandemic, the GH provocative tests of some children were delayed. When performed about 5 months after the first measurement (156 ± 87 days), 10 children were diagnosed to have ISS. Hence, this represents a control group of children. While all 31 children of the first group showed an increase in their growth rate after testing, in the 10 children whose testing was delayed, the growth rate increased slightly in 2, remained unchanged in 3, and decreased in 5 before the tests (Fig. 2A). The growth rate increased after the tests in all children. The median (IQR) 90 days growth of children at first, second observation before the tests and after the tests were 0.7 (0.46–0.99) cm, 0.6 (0.41─ 0.94) cm (*p* = 0.861) and 2.3 (1.9–3.7) cm (*p* = 0.001) respectively (Fig. 2B).

**Fig. 3:**
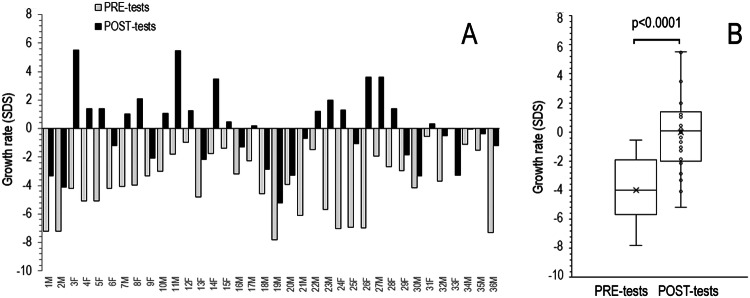
Effect of GH stimulation tests on growth rate of children with ISS. **A** individual estimated 90 days growth rate before tests time (PRE-tests) and after (POST-tests). **B** mean (x) median, quartiles, and individual values of growth rate. SDS, standard deviation scores. Variables were compared using Wilcoxon’s signed-rank test. Median, mean, SD, Q1, Q3 pre-test and post-test respectively: −4.0, −3.9, 2.1, −5.4, 2.1 and 0.1, 0.1, 2.6, −1.9, 1.4

The children’s growth rates before and after the tests were calculated also as standard deviation scores (SDS) considering sex and age. All children had a growth rate before the tests below the mean, ranging from −0.57─ −7.81 SDS (Fig. 3A). At the measurement after the tests, the growth rates of all the children had increased. Eighteen raised above the mean and eighteen increased remaining below the mean. The median (IQR) growth rate of children pre- and post-tests were −4.0 (−5.4–−2.1) SDS and 0.1 (−1.9–1.4) SDS respectively, a difference highly significant (*p* < 0.0001) (Fig. 3B).

**Fig. 4:**
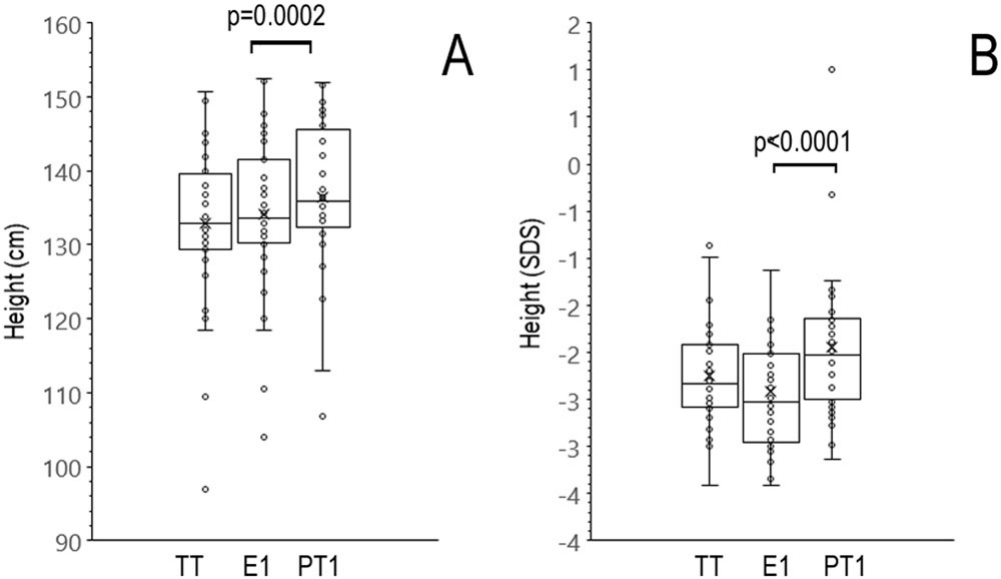
Effect of GH stimulation tests on the height of children with ISS. Effect of GH stimulation tests on the height of children with ISS. **A** mean (x), median, quartiles, and individual values of height of children at test time (TT), expected height after 180 days based on the pre-tests annual growth (E1), and real height measured 180 days after the tests (PT1). **B** mean, median, quartiles, and individual values of heights expressed as SDS. Variables were compared using the Wilcoxon’s signed-rank test

The median (IQR) height of children at tests time was 132.0 (129.3–139.0) cm, the expected median (IQR) height 180 days after the tests considering their pre-tests growth rate, was 132.9 (130.3–141.5) cm and the measured height 180 days after the tests was 135.5 (132.6–144.6) cm (p < 0.0001), a difference of 2.6 cm (Fig. 4A). Also, the final post-tests height calculated as SDS (−2.0 (−2.5–−1.6) was higher than expected height after 180 days considering their pre-tests annual growth rate (−2.5 (−3.0–−2.0), *p* < 0.0001) (Fig. 4B).

## Discussion

Diagnosis of GHD in children with short stature is based on the assessment of GH peak in two stimulation tests. However, a correct diagnosis remains a challenge because there is not a general agreement on the cut-off peak that distinguishes GHD from ISS. The Endocrine Society has adopted a 4.1 ng/mL peak GH [11, 12], whereas the GH Research Society and other medical societies, including the European Society of Endocrinology, suggest peak GH cut-off limits ranging from 4.2 to 11.5 ng/mL based on BMI [12–14]. In our study, all children enrolled displayed a GH peak above 8 ng/mL after the two stimulus tests, the cut-off chosen by the AIFA for rhGH therapy reimbursement. The idiopathic nature of the short stature of these children is supported also by the projected adult height based on the height of the parents. Indeed, the mid-parental height (172.4 ± 4.2 in boys, 158.3 ± 6.2 in girls) was below the Italian mean (176 cm in males, 163 cm in females), according to Cacciari growth charts for height [9].

Hereupon based on tests response, the enrolled children were considered to have ISS and left untreated. In the absence of evidence of the origin of the short stature, specific advice was not given, and the children’ss habits and diet remained unchanged during follow-up.

Despite that, an unexpected significant increase in the growth rate after the tests was shown by all children. In both females and males, mean growth normalized at 90 days increased from 0.7 cm prior to testing to 2.4 cm measured at an average time of 180 days after the tests, displaying a mean 3.4-fold increase.

It is remarkable that the growth rate of the control group of children with ISS after 5 months of observation before the tests did not change, while it increased after the tests.

Because a minimal change in the growth rate is expected with the age increase also at a short interval, we determined the growth rate as SDS. Even by this measurement, the mean growth rate increased from −4.0 before the tests to +0.3 after the tests. Thus, not only did the growth rate increase but it was even slightly higher than the mean of normal Italian children. It is noteworthy that, despite the children being of different genders and ages, all displayed a growth rate increase, with 18 of them rising above the mean. We searched by Spearman Rank for a correlation between increased growth rate and age or growth rate prior to testing without finding anything. Importantly, the growth observed in all patients was independent of pubertal development as both FSH, LH, total testosterone, and 17-beta estradiol levels and tanner pubertal stage remained pre-pubertal before and after the stimulus tests at the time of height measurement (mean 180 days).

In conclusion, these data indicate that two sequential somatotropic axis provocative tests increase the growth rate in prepubertal children with ISS and that this effect is not related to pubertal development. We do not have an explanation for this unexpected phenomenon. One possibility is a prolonged increase of GH after the second GH provocative test, or the tests could modify the GH secretion modality and alter the pulsatile GH secretion pattern. In men, pulsatile GH secretion occurs during the day, with a rhythmicity of about 2 h. Pulsatile GH secretion is considered important for its physiological effects. In hypophysectomised rats given continuous or pulsatile GH infusion, growth was stimulated maximally by pulsatile GH levels [15]. An alternative explanation is a prolonged IGF-1 increased production or an increased sensitivity of the growth plate to GH/IGF-1.

Initially, in fact, it was thought that IGF-1 was produced exclusively by the liver and its expression was completely dependent on GH [16]; subsequently, it was demonstrated that, in addition to the liver, IGF-1 is produced, together with GH, also by the same cells on which both hormones exert their action through autocrine and paracrine mechanisms [16].

GH, we know, has a direct effect on bone [16]; if added to chondrocyte cultures, it was able to promote its proliferation [17] that depends, in part, on the local and GH-mediated synthesis of IGF-1 [18], as shown by in vitro experiments on stimulated chondrocytes from GH, in which the simultaneous administration of anti-IGF-1 antibodies was able to block its clonal expansion [18]. Furthermore, GH and IGF-1 participate synergistically in the longitudinal growth in correspondence with the epiphyseal plate [16] with a pleiotropic action of the growth hormone documented by the expression of its receptor in all zones and with a more selective IGF-1 mechanism in the ossification process [19].

These hypotheses should be investigated by measuring GH and IGF-1 during follow-up after the tests. We have no data regarding these measurements so, we don’t know the duration of the stimulatory effect of the GH stimulus tests on the growth rate. The lack of an extended measurement of IGF-1 and GH after the tests, the characterization of the GH pulsatile secretion together with the small sample size, and the lack of evaluation of the bone age of the enrolled patients are limits of the study.

## References

[1] E.J. Velez, S. Unniappan, A comparative update on the neuroendocrine regulation of growth hormone in vertebrates. Front. Endocrinol. **11**, 614981 (2020) 10.3389/fendo.2020.614981 PMC7940767 33708174

[2] S. Loche, C. Bizzarri, M. Maghnie, A. Faedda, C. Tzialla, M. Autelli, M.R. Casini, M. Cappa, Results of early reevaluation of growth hormone secretion in short children with apparent growth hormone deficiency. J. Pediatr. **140**, 445–449 (2002) 12006959 10.1067/mpd.2002.122729

[3] J. Smyczynska, A. Lewinski, M. Hilczer, R. Stawerska, M. Karasek, Partial growth hormone deficiency (GHD) in children has more similarities to idiopathic short stature than to severe GHD. Endokrynol. Pol. **58**, 182–187 (2007) 17940982

[4] W.M. Drake, S.J. Howell, J.P. MonsonS, M. Shalet, Optimizing gh therapy in adults and children. Endocr. Rev. **22**, 425–450 (2001) 11493578 10.1210/edrv.22.4.0438

[5] L. Penta, M. Cofini, L. Lucchetti, L. Zenzeri, A. Leonardi, L. Lanciotti, D. Galeazzi, A. Verrotti, S, Esposito: Growth Hormone (GH) therapy during the transition period: should we think about early retesting in patients with idiopathic and isolated GH deficiency? Int. J. Environ. Res. Public Health **16**, 307–316 (2019) 10.3390/ijerph16030307 PMC6388362 30678118

[6] T. Stanley, Diagnosis of growth hormone deficiency in childhood. Curr. Opin. Endocrinol. Diab. Obes. **19**, 47–52 (2012) 10.1097/MED.0b013e32834ec952 PMC3279941 22157400

[7] C. Guzzetti, A. Ibba, S. Pilia, N. Beltrami, N. Di Iorgi, A. Rollo, N. Fratangeli, G. Radetti, S. Zucchini, M. Maghnie, M. Cappa, S. Loche, Cut-off limits of the peak GH response to stimulation tests for the diagnosis of GH deficiency in children and adolescents: study in patients with organic GHD. Eur. J. Endocrinol. **175**, 41–47 (2016) 27147639 10.1530/EJE-16-0105

[8] G. Saggese, M.B. Ranke, P. Saenger, R.G. Rosenfeld, T. Tanaka, J.L. Chaussain, M.O. Savage, Diagnosis and treatment of growth hormone deficiency in children and adolescents: towards a consensus. Ten years after the Availability of Recombinant Human Growth Hormone Workshop held in Pisa, Italy, 27–28 March 1998. Horm. Res. **50**, 320–340 (1998) 9973672 10.1159/000023298

[9] E. Cacciari, S. Milani, A. Balsamo, E. Spada, G. Bona, L. Cavallo, F. Cerutti, L. Gargantini, N. Greggio, G. Tonini, A. Cicognani, Italian cross-sectional growth charts for height, weight and BMI (2 to 20 yr). J. Endocrinol. Investig. **29**, 581–593 (2006) 16957405 10.1007/BF03344156

[10] Growth Hormone Research S., Consensus guidelines for the diagnosis and treatment of growth hormone (GH) deficiency in childhood and adolescence: summary statement of the GH Research Society. GH Research Society. J. Clin. Endocrinol. Metab. **85**, 3990–3993 (2000) 11095419 10.1210/jcem.85.11.6984

[11] M.E. Molitch, D.R. Clemmons, S. Malozowski, G.R. Merriam, S.M. Shalet, M.L. Vance; S. Endocrine Society’s Clinical Guidelines, P.A. Stephens, Evaluation and treatment of adult growth hormone deficiency: an Endocrine Society Clinical Practice Guideline. J. Clin. Endocrinol. Metab. **91**, 1621–1634 (2006) 16636129 10.1210/jc.2005-2227

[12] D.M. Cook, K.C. Yuen, B.M. Biller, S.F. Kemp, M.L. Vance; E. American Association of Clinical, American Association of Clinical Endocrinologists medical guidelines for clinical practice for growth hormone use in growth hormone-deficient adults and transition patients - 2009 update. Endocr. Pr. **15**(Suppl 2), 1–29 (2009) 10.4158/EP.15.S2.1 20228036

[13] F. Bogazzi, L. Manetti, M. Lombardi, C. Giovannetti, V. Raffaelli, C. Urbani, I. Scattina, P. Pepe, A. Iannelli, E. Martino, G. Rossi, Impact of different cut-off limits of peak GH after GHRH-arginine stimulatory test, single IGF1 measurement, or their combination in identifying adult patients with GH deficiency. Eur. J. Endocrinol. **164**, 685–693 (2011) 21307143 10.1530/EJE-10-1068

[14] K.K. Ho; G.H.D.C.W. Participants, Consensus guidelines for the diagnosis and treatment of adults with GH deficiency II: a statement of the GH Research Society in association with the European Society for Pediatric Endocrinology, Lawson Wilkins Society, European Society of Endocrinology, Japan Endocrine Society, and Endocrine Society of Australia. Eur. J. Endocrinol. **157**, 695–700 (2007) 18057375 10.1530/EJE-07-0631

[15] G. Aimaretti, C. Baffoni, S. Bellone, L. Di Vito, G. Corneli, E. Arvat, L. Benso, F. Camanni, E. Ghigo, Retesting young adults with childhood-onset growth hormone (GH) deficiency with GH-releasing-hormone-plus-arginine test. J. Clin. Endocrinol. Metab. **85**, 3693–3699 (2000) 11061526 10.1210/jcem.85.10.6858

[16] J. Devesa, C. Almenglo, P. Devesa, Multiple effects of growth hormone in the body: is it really the hormone for growth? Clinical medicine insights. Endocrinol. Diab. **9**, 47–71 (2016) 10.4137/CMED.S38201 PMC5063841 27773998

[17] A. Lindahl, J. Isgaard, A. Nilsson, O.G. Isaksson, Growth hormone potentiates colony formation of epiphyseal chondrocytes in suspension culture. Endocrinology **118**, 1843–1848 (1986) 3698898 10.1210/endo-118-5-1843

[18] C. Jux, K. Leiber, U. Hugel, W. Blum, C. Ohlsson, G. Klaus, O. Mehls, Dexamethasone impairs growth hormone (GH)-stimulated growth by suppression of local insulin-like growth factor (IGF)-I production and expression of GH- and IGF-I-receptor in cultured rat chondrocytes. Endocrinology **139**, 3296–3305 (1998) 9645706 10.1210/endo.139.7.6099

[19] E.A. Parker, A. Hegde, M. Buckley, K.M. Barnes, J. Baron, O. Nilsson, Spatial and temporal regulation of GH-IGF-related gene expression in growth plate cartilage. J. Endocrinol. **194**, 31–40 (2007) 17592018 10.1677/JOE-07-0012

